# Interleukin-37 Enhances the Suppressive Activity of Naturally Occurring CD4^+^CD25^+^ Regulatory T Cells

**DOI:** 10.1038/srep38955

**Published:** 2016-12-12

**Authors:** Da-Wei Wang, Ning Dong, Yao Wu, Xiao-Mei Zhu, Chun-Ting Wang, Yong-Ming Yao

**Affiliations:** 1 Department of Critical Care Medicine, Shandong Provincial Hospital Affiliated to Shandong University, Jinan 250101, P. R.China; 2 Trauma Research Center, First Hospital Affiliated to the Chinese PLA General Hospital, Beijing 100048, P. R.China; 3 State Key Laboratory of Kidney Disease, the Chinese PLA General Hospital, Beijing 100853, P. R.China

## Abstract

Naturally occurring CD4^+^CD25^+^ regulatory T cells (Tregs) are essential for the suppression of autoimmunity and can control the immune-mediated pathology during the early phase of sepsis. Our previous data showed that silencing interleukin-37 (IL-37) in human CD4^+^CD25^+^ Tregs obviously reduced the suppressive activity of CD4^+^CD25^+^ Tregs. Here, we found that rhIL-37 stimulation markedly enhanced the suppressive activity of CD4^+^CD25^+^ Tregs isolated from naive C57BL/6 J mice in the absence or presence of lipopolysaccharide (LPS). Treatment with rhIL-37 could significantly upregulate the expression of cytotoxic T-lymphocyte-associated antigen (CTLA)-4 and forkhead/winged helix transcription factor p3 (Foxp3) on CD4^+^CD25^+^ Tregs. Also, rhIL-37 stimulation promoted the production of transforming growth factor-β1 (TGF-β1) but not IL-10 in the supernatants of cultured CD4^+^CD25^+^ Tregs. Pretreated CD4^+^CD25^+^ Tregs with rhIL-37 in the presence or absence of LPS were cocultured with CD4^+^CD25^−^ T cells, ratio of IL-4/interferon-γ in the supernatants obviously increased in IL-37-stimulated groups. In addition, early administration of IL-37 significantly improved the survival rate of septic mice induced by cecal ligation and puncture. Taken together, we concluded that rhIL-37 enhances the suppressive activity of CD4^+^CD25^+^ Tregs and might be a potential immunomodulator for the treatment of septic complications.

There is a growing body of evidence to show that naturally occurring CD4^+^CD25^+^ regulatory T cells (Tregs) are crucial to the proper maintenance of immune self-tolerance and homeostasis^1^. CD4^+^CD25^+^ Tregs constitutively express forkhead/winged helix transcription factor p3 (Foxp3), glucocorticoid-induced tumor necrosis factor (TNF) receptor (GITR), and cytotoxic T-lymphocyte-associated antigen (CTLA)-4 2,3,4. Mice lacking these key immunoregulatory molecules will exhibit lethal lymphoproliferative phenotypes. Both human and murine CD4^+^CD25^+^ Tregs express Toll-like receptor 4 (TLR4), which can be activated by lipopolysaccharide (LPS)^5^. Subsequently, survival and suppressive activity of Tregs was maintained and enhanced. It has been demonstrated that Tregs can produce various immunosuppressive cytokines [e.g. transforming growth factor (TGF)-β, interleukin (IL)-10 and IL-35], which contribute to inhibition of effector T cells^6^,^7^,^8^. Functionally, Tregs show a marked hypoproliferation and can obviously suppress polyclonal T cell activation *in vitro* by inhibiting IL-2 production^9^. Furthermore, Tregs deplete IL-2 through binding to their constitutively highly expressed IL-2 receptor. Then T effect cells, which are deprived from IL-2, undergo apoptosis^10^. Tregs can also control helper T cell (Th)1, Th2 or Th17 type immune responses by modifying their expression of transcription factors, which include Th1-specifying transcription factor T-bet, interferon (IFN) regulatory factor-4 (IRF4), and signal transducer and activator of transcription 3 (STAT3)^11^,^12^,^13^.

Sepsis is defined as life-threatening organ dysfunction caused by a dysregulated immune response to infection^14^. Several studies suggested that Tregs are beneficial to control the immune response in the early phase of sepsis. For instance, adoptive transfer of CD4^+^CD25^+^ Tregs before or at 6 hours after the induction of cecal ligation and puncture (CLP) had a protective effect on animal survivals^15^. During the early hyper-inflammatory phase of sepsis, specific depletion of Foxp3^+^ Tregs leads to a more severe course of sepsis with a higher mortality rate and a significantly higher IL-6 level^16^. In contrast, depletion of Tregs was detrimental to survival from LPS-induced acute inflammation and *Escherichia coli* infection^17^. However, Tregs are detrimental to septic host in certain conditions. For instance, augmented levels of Tregs inversely were correlated with lymphocyte proliferation, thereby contributing to the immune paralysis in the setting of sepsis^18^. Sepsis-induced expansion and enhanced function of Tregs could create an environment to potentiate tumor growth and impair the antitumor response^19^. On the contrary, reducing function of Tregs using GITR antibody restored CD4^+^ T cell proliferation, decreased the counts of bacteria in spleen, and improved the outcome of septic animals^20^.

IL-37, formerly referred to as IL-1F7, is transcribed as five different splice variants (IL-37a-e). It is exclusively expressed in human cells, but not detected in mice^21^. Interestingly, human IL-37 exhibits effects on murine cells that are comparable to those on human cells. It has been reported that IL-37 could down-regulate innate immunity and adaptive immunity. For example, expression of IL-37 in macrophages or epithelial cells suppressed production of pro-inflammatory cytokines, whereas the abundance of these cytokines increased with silencing of endogenous IL-37 in human blood cells^22^. Similarly, rhIL-37 inhibited murine neutrophil activation or LPS-induced production of pro-inflammatory cytokines in murine Kupffer cells^23^. The induction of IL-37 expression in dendritic cells (DCs) negatively modulated DC maturation and function, thereby generating semimature tolerogenic DCs to impair the activation of T effector cells and favor the development of Tregs^24^. Moreover, *in vivo* studies showed that IL-37 played an anti-inflammatory role in various inflammatory diseases. Transgenic mice overexpressing human IL-37 were protected from endotoxemia, concanavalin A-induced hepatitis, dextran sulfate sodium-induced colitis, myocardial, cerebrial and hepatic ischemia/reperfusion injury^23^,^25^,^26^,^27^,^28^,^29^.

As we have recently shown, silencing IL-37 in human CD4^+^CD25^+^ Tregs obviously reduced the suppressive activity of CD4^+^CD25^+^ Tregs^30^. In the current study, the aims were to determine whether recombinant human IL-37 (rhIL-37) enhanced the suppressive activity of CD4^+^CD25^+^ Tregs in the presence or absence of LPS. Concomitantly, expression of CTLA-4 and Foxp3, production of IL-10 and TGF-β, ability of depleting IL-2, and effect of Th2-cell polarization were also assessed after rhIL-37 stimulation. In addition, the potential effects of treatment with rhIL-37 on the outcome were evaluated in mice subjected to septic challenge.

## Results

### The effect of IL-37 on suppressive activity of CD4^+^CD25^+^ Tregs

**Figure 1:**
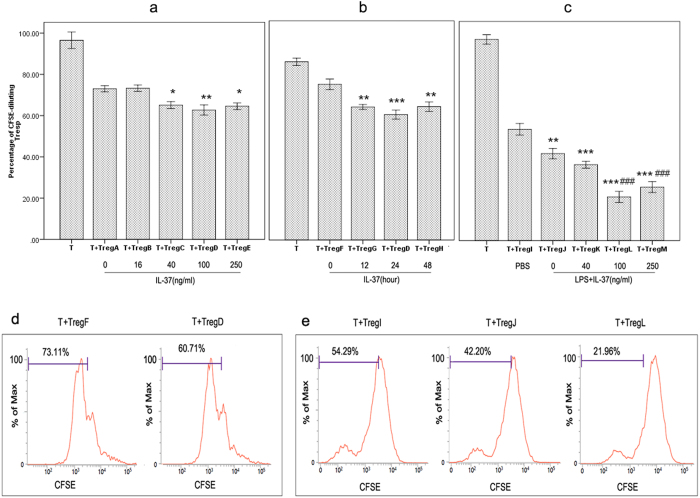
The effect of IL-37 on the suppressive activity of CD4^+^CD25^+^ Tregs. CD4^+^CD25^+^ Tregs were mixed with CFSE-labeled CD4^+^CD25^−^ T cells (responder T cells, Tresp) in a ratio of 1:4, and the proliferation of Tresp was assessed by the percentage of CFSE diluting Tresp. n = 5 per group. (**a**) Tregs A, B, C, D, and E were pre-stimulated with PBS and IL-37 (16, 40, 100 and 250 ng/ml) respectively for 24 hours. *P < 0.05, **P < 0.01 vs. Treg A group. (**b**) Tregs F, G, D, and H were pre-treated with PBS for 12 hours or 100 ng/ml IL-37 for 12, 24, and 48 hours, respectively. **P  < 0.01, ***P < 0.001 vs. Treg F group. Percentage of CFSE diluting Tresp were shown in (**d**). (**c**) Treg I was pre-stimulated with PBS for 3 days; Treg J, K, L, and M were pre-stimulated with LPS (5 μg/ml) for 3 days. At 24 hours before the end of pre-stimulation, IL-37 (40 ng/ml, 100 ng/ml and 250 ng/ml) was respectively added to the culture of Tregs K, L, and M. **P  < 0.01, ***P < 0.001 vs. Treg I group. ^###^P < 0.001 versus Treg J group. Percentage of CFSE diluting Tresp were shown in Fig. 1e.

CD4^+^CD25^+^ Tregs pre-treated with IL-37 in increasing concentrations for 24 hours were mixed with CD4^+^CD25^−^ T cells at a ratio of 1:4. The effect of IL-37 on T lymphocyte proliferation was shown in Fig. 1a. There was a decrease in proliferation of CD4^+^CD25^−^ T cells when cocultured with CD4^+^CD25^+^ Tregs. Proliferative activity of T lymphocytes was further decreased when cocultured with IL-37 stimulated CD4^+^CD25^+^ Tregs, especially in 100 ng/ml group (Treg D group). Thus, IL-37 augmented the ability of CD4^+^CD25^+^ Tregs to suppress T cells, and such response was most significant in 24-hour group (Treg D group) when stimulated with IL-37 for different time points (Fig. 1b and d). LPS can activate murine Tregs and enhance suppressive activity of Tregs. To confirm whether IL-37 could regulate the suppressive activity of CD4^+^CD25^+^ Tregs in the presence of LPS, CD4^+^CD25^+^ Tregs were pre-incubated with LPS plus IL-37 before the suppression assay. The results showed that LPS markedly induced enhancement of suppression of CD4^+^CD25^+^ Tregs, which was more obvious after LPS plus IL-37 stimulation (Fig. 1c and e). In other words, IL-37 could enhance the suppressive activity of CD4^+^CD25^+^ Tregs in the presence of LPS.

### Effects of IL-37 on CTLA-4 and Foxp3 expressions in CD4^+^CD25^+^ Tregs

**Figure 2:**
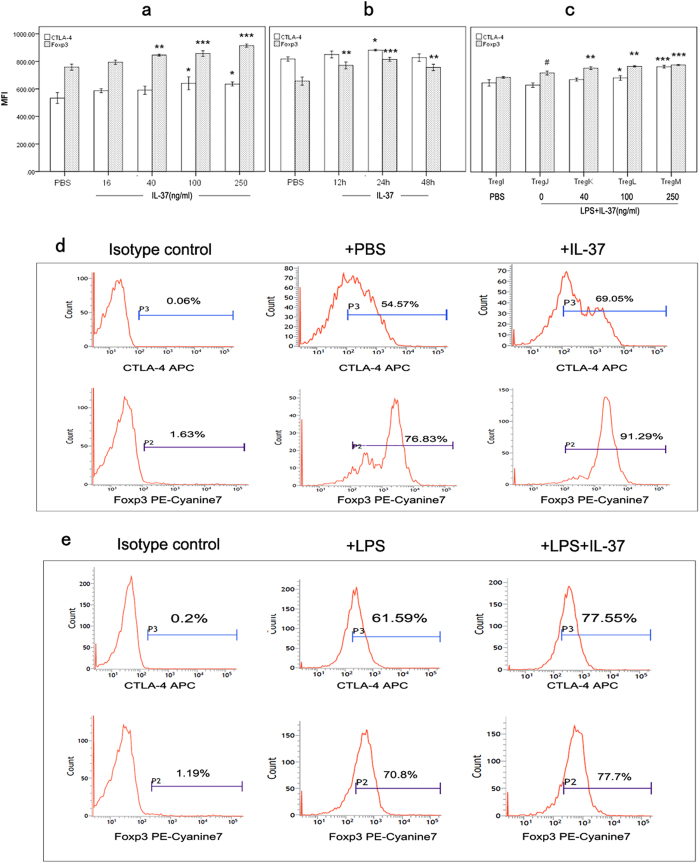
Effects of IL-37 on CTLA-4 and Foxp3 expressions in CD4^+^CD25^+^ Tregs. (**a**) CD4^+^CD25^+^ Tregs were stimulated with PBS and IL-37 (16, 40, 100 and 250 ng/ml) respectively. After 24 hours of incubation, the expression of CTLA-4 and Foxp3 was determined by flow cytometry. *P < 0.05, **P < 0.01, ***P < 0.001 vs. PBS group (n = 4). **(b)** CD4^+^CD25^+^ Tregs were treated with PBS for 12 hours or 100 ng/ml IL-37 for 12, 24, and 48 hours respectively. Then expressions of CTLA-4 as well as Foxp3 were analyzed. *P < 0.05, **P  < 0.01, ***P < 0.001 vs. PBS group. n = 4. Representative histograms of CTLA-4 and Foxp3 expressions were shown in (**d**) (**c**) Treg I was pre-treated with PBS for 3 days; Tregs J, K, L, and M were pre-stimulated with LPS (5 μg/ml) for 3 days. At 24 hours before the end of pre-stimulation, IL-37 (40 ng/ml, 100 ng/ml and 250 ng/ml) was respectively added to the culture of Tregs K, L, and M. After treatment, Tregs were used to determine expressions of CTLA-4 and Foxp3. *P < 0.05, **P  < 0.01, ***P < 0.001 vs. Treg J group. ^#^P < 0.05 vs. Treg I group. n = 4. Representative histograms of CTLA-4 and Foxp3 expression were shown in Fig. 2e.

Effects of IL-37 in different concentrations on expressions of CTLA-4 and Foxp3 were determined by flow cytometry after 24-hour incubation of CD4^+^CD25^+^ Tregs (Fig. 2a). IL-37 stimulation for 24 hours significantly increased Foxp3 expression ranging from 40 ng/ml to 250 ng/ml, and increased CTLA-4 expression ranging from 100 ng/ml to 250 ng/ml. When using 100 ng/ml IL-37 to stimulate CD4^+^CD25^+^ Tregs, IL-37-mediated responses for CTLA-4 and Foxp3 expression were more significant in 24-hour group (Fig. 2b). Representative histograms of CTLA-4 and Foxp3 expressions were shown in Fig. 2d. Meanwhile, we examined the effects of IL-37 on CTLA-4 and Foxp3 expression on CD4^+^CD25^+^ Tregs in the presence of LPS stimulation. As shown in Fig. 2c, Foxp3 expression but not CTLA-4 expression was upregulated when CD4^+^CD25^+^ Tregs were stimulated with LPS alone (Treg J group vs. Treg I group). In addition, IL-37 could obviously upregulate CTLA-4 and Foxp3 expressions in the presence of LPS stimulation. Representative histograms of CTLA-4 and Foxp3 expressions were shown in Fig. 2e.

### Effects of IL-37 on production of TGF-β1 and IL-10 in CD4^+^CD25^+^ Tregs

**Figure 3:**
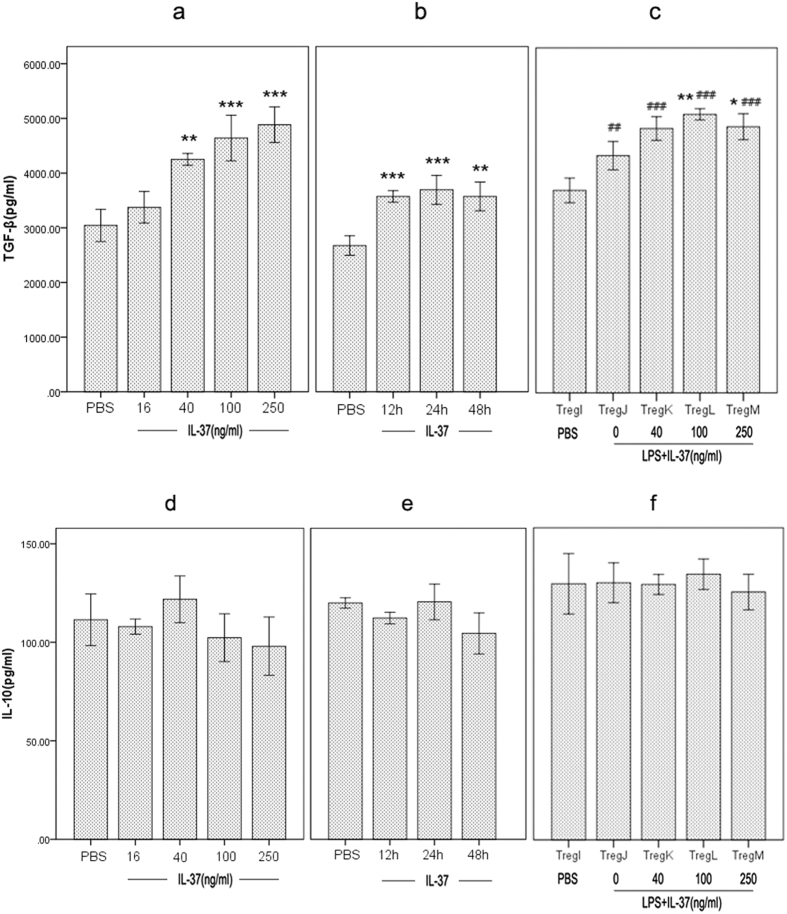
Effect of IL-37 on production of TGF-β1 and IL-10 in CD4^+^CD25^+^ Tregs. (**a**) and (**d**) CD4^+^CD25^+^ Tregs were treated with IL-37 (16, 40, 100 and 250 ng/ml). After 24 hours of incubation, the production of TGF-β1 and IL-10 was determined by Enzyme-linked immunosorbent assay (ELISA). **P < 0.01, ***P < 0.001 vs. PBS group (n = 6). (**b**) and (**e**) CD4^+^CD25^+^ Tregs were treated with 100 ng/ml IL-37, and the production of TGF-β1 and IL-10 was analyzed at different time points. **P < 0.01, ***P < 0.001 vs. PBS group (n = 6). (**c**) and (**f**) Treg I was treated with PBS for 3 days; Treg J, K, L, and M were treated with LPS (5 μg/ml) for 3 days. At 24 hours before the end of LPS treatment, IL-37 (40 ng/ml, 100 ng/ml and 250 ng/ml) was respectively added to the culture of Treg K, L, and M. Then levels of TGF-β1 and IL-10 were analyzed. *P < 0.05, **P < 0.01 vs. Treg J group. ^##^P < 0.01, ^###^P < 0.001 vs. Treg I group. n = 6.

Since TGF-β and IL-10 are cytokines with potent anti-inflammatory activities that is produced by CD4^+^CD25^+^ Tregs, we next determined the effect of IL-37 on production of TGF-β1 as well as IL-10 in CD4^+^CD25^+^ Tregs. Upon IL-37 treatment, secretion of TGF-β1 by CD4^+^CD25^+^ Tregs was prominently enhanced compared to the un-stimulated group (Fig. 3a and b). Similarly, treatment with LPS enhanced the secretion of TGF-β by CD4^+^CD25^+^ Tregs. In the condition of LPS stimulation, IL-37 further enhanced the production of TGF-β by CD4^+^CD25^+^ Tregs (Fig. 3c). However, IL-37 stimulation or LPS plus IL-37 stimulation had no influence on the release of IL-10 by CD4^+^CD25^+^ Tregs (Fig. 3d,e and f).

### Effects of IL-37 on IL-2, IL-4, and IFN-γ production in CD4^+^CD25^−^ T cells in coculture experiments

**Figure 4:**
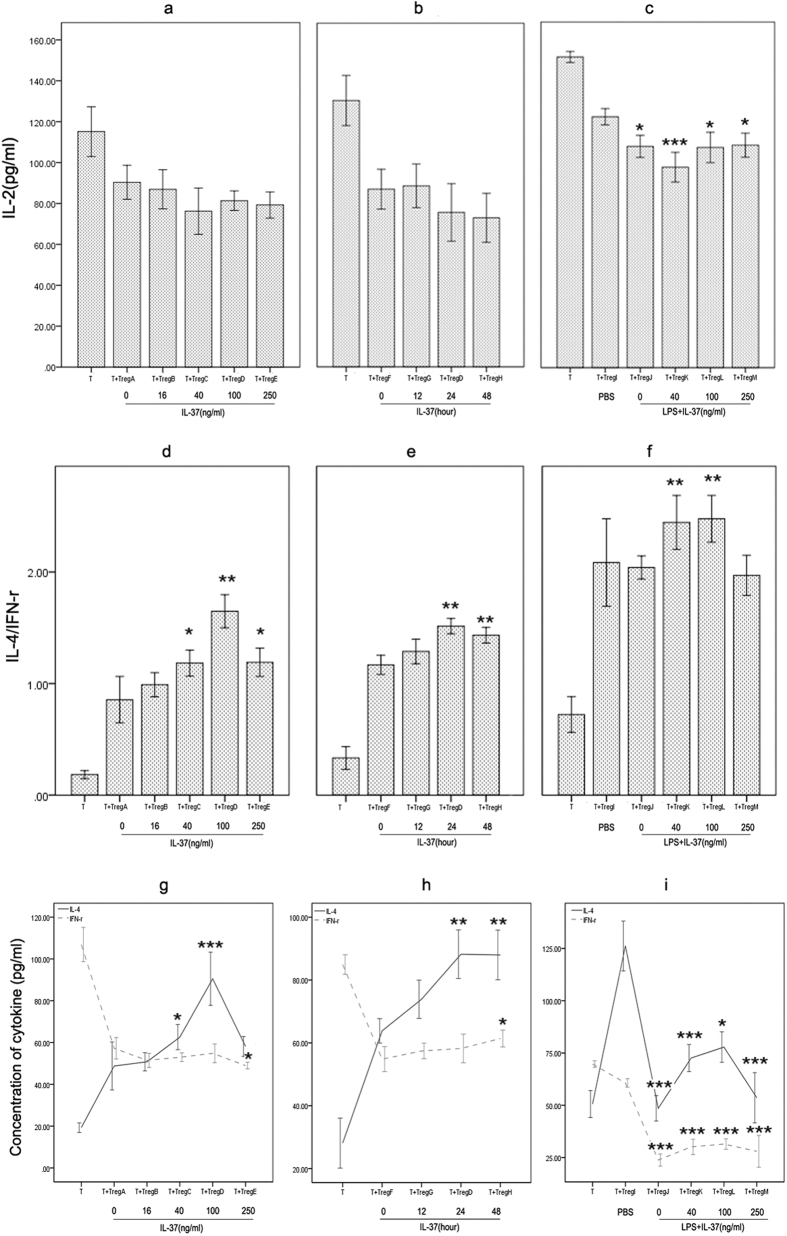
Effects of IL-37 on the release of IL-2, IL-4, and IFN-γ in CD4^+^CD25^−^ T cells in coculture experiments. Pre-stimulated CD4^+^CD25^+^ Tregs were mixed with CD4^+^CD25^−^ T cells. After 96 hours, levels of IL-2, IL-4, and IFN-**γ** in the conditioned media were determined by ELISA. n = 6 per group. (**a,d** and **g**) Tregs A, B, C, D, and E were pre-treated with PBS and IL-37 (16, 40, 100 and 250 ng/ml) respectively for 24 hours. *P < 0.05, **P < 0.01, ***P < 0.001 vs. Treg A group. (**b,e** and **h**) Tregs F, G, D, and H were pre-treated with PBS for 12 hours or 100 ng/ml IL-37 for 12, 24, and 48 hours, respectively. *P < 0.05, **P < 0.01 vs. Treg F group. (**c,f** and **i**) Treg I was pre-treated with PBS for 3 days; Tregs J, K, L, and M were pre-treated with LPS (5 μg/ml) for 3 days. At 24 hours before the end of pretreatment, IL-37 (40 ng/ml, 100 ng/ml and 250 ng/ml) was respectively added to the culture of Tregs K, L, and M. *P < 0.05, **P < 0.01, ***P < 0.001 vs. Treg I group.

CD4^+^CD25^+^ Tregs pre-treated with IL-37 were mixed with CD4^+^CD25^−^ T cells at a ratio of 1:4. After 96 hours, supernatants were harvested for analysis of cytokine production of CD4^+^CD25^−^ T cells. The effect of IL-37 on IL-2 production was shown in Fig. 4a and b. Consistent suppression of IL-2 was observed in various Treg groups, but no significant difference in IL-2 level was noted between normal Treg group and IL-37 stimulated Treg groups. Interestingly, compared to normal Treg group, suppression of IL-2 was enhanced when CD4^+^CD25^+^ Tregs were pre-stimulated with LPS (Fig. 4c). That is to say, LPS markedly induced enhancement of Tregs’ ability depleting IL-2. However, no significant difference in IL-2 level between LPS stimulated group and LPS plus IL-37 stimulated group. We also investgated the effect of IL-37 on the T-cell polarization in response to CD4^+^CD25^+^ Tregs. As shown in Fig. 4d and e, CD4^+^CD25^+^ Tregs efficiently induced Th2-cell polarization as demonstrated by an increase of IL-4/IFN-γ ratio, which is an indicator of Th1/Th2 balance. Moreover, IL-37 stimulation enhanced the effect of CD4^+^CD25^+^ Tregs induced Th2-cell polarization, no matter with or without LPS stimulation (Fig. 4d,e and f). Absolute values of IL-4 and IFN-γ were shown in Fig. 4g–i corresponding to Fig. 4d–f.

### The effect of IL-37 on septic animal survival

**Figure 5:**
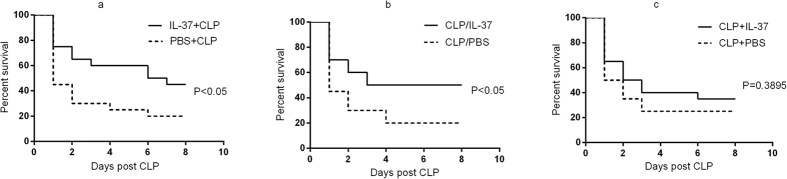
Effect of IL-37 on survival rate of septic animals. CLP mice were given an i.p. injection of 1 μg IL-37 at 2 hours prior to surgery, 2 hours post surgery, or immediately after surgery. The control groups were injected an identical volume (200 μl) of PBS intraperitoneally at the corresponding time point, which IL-37 was given. Animals were observed for up to 8 days to evaluate survival rates. n = 20 mice per group. **(a)** Survival curves of IL-37+CLP group and PBS+CLP group; **(b)** Survival curves of CLP/IL-37 group and CLP/PBS group; **(c)** Survival curves of CLP+IL-37 group and CLP+PBS group.

In endotoxemia mice, pre-treatment with IL-37 reduced circulating and organ cytokine levels, and alleviated LPS-induced weight loss and hypothermia. Here, we produced polymicrobial sepsis by CLP procedure and evaluated whether rhIL-37 had a protective effect on septic animal survival. Compared with the PBS control group, pre-treatment or treatment immediately after CLP with IL-37 significantly improved survival rate (P < 0.05; Fig. 5a and b). However, IL-37 administration at 2 hours post CLP did not affect mortality of the CLP model (Fig. 5c). These results indicated that early administration of IL-37 could improve the outcome of septic animals.

## Discussion

Our findings demonstrated that rhIL-37 could obviously enhance the suppressive activity of CD4^+^CD25^+^ Tregs isolated from naive C57BL/6 J mice in the absence or presence of LPS. Treatment with IL-37 upregulated the expression of CTLA-4 and Foxp3 on CD4^+^CD25^+^ Tregs, and promoted the production of TGF-β1 by CD4^+^CD25^+^ Tregs. Moreover, IL-37 stimulation markedly enhanced CD4^+^CD25^+^ Treg-mediated response of Th2-cell polarization in the absence or presence of LPS. In addition, it was noted that early administration of rhIL-37 had a protective effect on survivals of septic animals.

There is a growing body of evidence to show that Tregs play an important role in the maintenance of immunological self-tolerance and the modulation of immune responses. Increase in the numbers or functional activity of Tregs might be beneficial in the treatment of autoimmune diseases, allergic diseases, allograft rejection as well as graft versus host disease (GVHD)^31^,^32^,^33^,^34^. In the present study, treatment with IL-37 significantly enhanced CD4^+^CD25^+^ Treg-mediated suppression of the proliferation of CD4^+^CD25^−^ T cells. Thus, we speculate that IL-37 might be useful for the treatment of autoimmune disease, allergy as well as allograft rejection and GVHD in Treg-targeted strategy. In accordance with such opinion, several studies have shown that IL-37 could restrain autoimmune diseases. It was found that IL-37 showed immunosuppressive response in patients with rheumatoid arthritis (RA) and in mice with collagen-induced arthritis (CIA) via suppressing IL-17 and IL-17–triggering cytokine production and limiting Th17 cell proliferation^35^. IL-37 also significantly inhibited the release of TNF-α, IL-1β, and IL-6 in peripheral blood mononuclear cells (PBMCs) of patients with systemic lupus erythematosus (SLE) *in vitro*^36^. In addition, the immunosuppression of IL-37 was reported in psoriasis, ankylosing spondylitis, and Graves’ disease^37^,^38^,^39^.

Here we noticed that stimulation with LPS activated CD4^+^CD25^+^ Tregs, which included upregulating the expression of Foxp3, increasing the production of TGF-β1, and enhancing the ability depleting IL-2. These findings were accordance with the previous study, which LPS could activate CD4^+^CD25^+^ Tregs through their expression of TLR4^5^,^40^,^41^. It has been demonstrated that LPS-activated Tregs inhibit reactive oxygen intermediates and cytokine production by neutrophils *in vitro*^40^. So we favor the idea that LPS-activated CD4^+^CD25^+^ Tregs might be beneficial in the reduction of harmful effects of excessive inflammation from the beginning of invasion of Gram-negative bacteria into the organism^41^. In the current study, we found that IL-37 stimulation upregulated the suppressive activity of CD4^+^CD25^+^ Tregs in the presence of LPS. Thus, IL-37-activated CD4^+^CD25^+^ Tregs might be advantageous to control the early inflammatory response in sepsis *in vivo*. This concept is supported by the results that early administration of rhIL-37 could obviously improve the outcome of septic animals.

Foxp3 is the most reliable marker for Tregs and determines the development and function of Tregs. Mutations of Foxp3 in humans results in deficiency or dysfunction of Tregs, and thus cause immune dysregulation, polyendocrinopathy, enteropathy, X-linked (IPEX) syndrome^42^. Ectopic expression of Foxp3 in conventional T cells leads the cells to become CD4^+^CD25^+^ Treg-like cells with suppressive activity on peripheral CD4^+^CD25^−^ T cells^2^. We found that upregulation of Foxp3 expression on CD4^+^CD25^+^ Tregs occurred in the presence of IL-37 stimulation. It indicates that IL-37-mediated enhancement of suppression of CD4^+^CD25^+^ Tregs is closely related to the upregulation of Foxp3 expression.

CTLA-4-dependent suppression is also crucial for Tregs in maintaining immunological self-tolerance and immune homeostasis. Treg-specific CTLA-4 deficiency impairs both *in vivo* and *in vitro* suppressive function of Tregs. A specific deficiency of CTLA-4 in Tregs results in spontaneous development of systemic lymphoproliferation, fatal T cell-mediated autoimmune disease, and hyperproduction of immunoglobulin E in mice, and it also produces potent tumor immunity^43^. In the present study, treatment with IL-37 markedly enhanced CTLA-4 expression on CD4^+^CD25^+^ Tregs in parallel with the upregulation of suppressive activity of CD4^+^CD25^+^ Tregs. Thus, increased expression of CTLA-4 might result in the enhanced suppressive effect of CD4^+^CD25^+^ Tregs in response to IL-37. Additionally, trend of CTLA-4 expression was similar to Foxp3 expression in the presence of IL-37, and both of them were upregulated simultaneously. These data indicated that there was a close relationship between CTLA-4 and Foxp3. In fact, Foxp3 directly controls CTLA-4 genes in CD4^+^CD25^+^ Tregs via binding to the promoter region of the CTLA-4 gene^44^.

Several anti-inflammatory cytokines, including IL-10 and TGF-β, are mainly implicated in the suppression of Tregs *in vivo* and the importance of *in vitro* remains controversial. IL-10-deficient Tregs failed to suppress mucosal immunity in a mouse model of inflammatory bowel disease, and blockade of IL-10R could abolish Treg-mediated inhibition of colitis^7^,^45^. Similarly, TGF-β dependency of suppression of Tregs was shown in murine models of colitis, diabetes and antitumor immunity^6^,^46^,^47^. Here, along with the increased formation of TGF-β1, the suppressive activity mediated by CD4^+^CD25^+^ Tregs was significantly enhanced in IL-37 stimulated-Tregs groups. Therefore, TGF-β1 appears to be involved in upregulating suppression of CD4^+^CD25^+^ Tregs induced by IL-37. However, the level of IL-10 in the culture supernatants of CD4^+^CD25^+^ Tregs did not change after IL-37 stimulation. Thus, we speculate that IL-10 might not be involved in the IL-37-induced suppression of CD4^+^CD25^+^ Tregs.

Tregs compete with effector T cells for IL-2 and cause cytokine deprivation-induced apoptosis of responder T cells^10^. With this in mind, we determined IL-2 levels in supernatants from CD4^+^CD25^+^ Tregs/CD4^+^CD25^−^ T cell cocultures. It was noted no difference in IL-2 levels between IL-37-stimulated group and un-stimulated group, which suggested that IL-2 secretion might be irrelevant to IL-37 induced suppressive activity of CD4^+^CD25^+^ Tregs. As we known, Tregs can control Th1, Th2 or Th17 type immune responses by modifying their expression of transcription factors, which include T-bet, IRF4, and STAT3^11^,^12^,^13^. Here we focused on Th1/Th2 balance and assessed whether IL-37 had influence on the Treg-mediated Th1/Th2 balance. We found that co-culture of CD4^+^CD25^−^ T cells and IL-37-stimulated CD4^+^CD25^+^ Tregs for 4 days resulted in an increase toward Th2 cytokine pattern in T-cell polarization, as indicated by elevation of IL-4/IFN-γ ratios. In view of the findings, it is our belief that IL-37 might play a significant role in the cell drifting toward Th2 in the CD4^+^CD25^+^ Treg-mediated immunosuppression.

In the present study, we mainly confirmed the effects of rhIL-37 stimulation on suppressive activity of CD4^+^CD25^+^ Tregs isolated from naive C57BL/6 J mice *in vitro*. The impact of treatment with rhIL-37 on suppressive activity of human CD4^+^CD25^+^ Tregs needs to be explored. Except for CD4^+^ T cells, Tregs can inhibit effector response mediated by CD8^+^ T cells and innate immune cells^6^,^40^,^47^. Suppressive activity of Tregs toward CD8^+^ T cells and innate immune cells needs to be elaborated in future. Moreover, the mechanism underlying nTregs-mediated immunosuppression includes multiple aspects. We did not investigate the expression of GITR, lymphocyte activation gene-3 (LAG-3), perforin, granzyme and programmed cell death ligand 1 (PD-L1) on Tregs^3^,^48^,^49^,^50^,^51^. Whether IL-37 influences these suppressive molecular or not remains unclear. In addition, the potential mechanism underlying survival protective effect to septic animals treated by IL-37 is complicated. *In vivo* effects of IL-37 administration on CD4^+^CD25^+^ Tregs, CD4^+^ T cells, dendritic cells, and macrophages in septic animals are largely unknown and should be further investigated. An experiment using Treg-depleted mice should be performed to clarify whether the protective effect of IL-37 is due to an action on CD4^+^CD25^+^ Tregs.

Nonetheless, the present study indicates that rhIL-37 can markedly upregulate the expression of CTLA-4/Foxp3 and increase production of TGF-β1. These alterations might lead to enhancement of CD4^+^CD25^+^ Treg-mediated suppression. Based on the important role of IL-37 in the suppressive activity of CD4^+^CD25^+^ Tregs, it may be feasible to use IL-37 as an immunotherapy for autoimmune diseases, allergies, allograft rejection or even sepsis.

## Materials and Methods

### Animals and reagents

Six-week-old male C57BL/6 J mice were obtained from the Institute of Laboratory Animal Science, Chinese Academy of Medical Sciences, and Peking Union Medical College, Beijing, China. Animals were housed in separate cages in a temperature-controlled room on a 12-hour light/dark cycle and allowed to acclimatize for at least 7 days before being used. All animals had free access to water but were fasted overnight before the experiment. All experimental manipulations were in accordance with the National Institutes of Health Guide for the Care and Use of Laboratory Animals, with the approval of the Scientific Investigation Board of the Chinese PLA General Hospital, Beijing, China.

Recombinant human IL-37b/IL-1F7b (7585-IL) was purchased from R&D Systems, Minneapolis, MN. LPS (L4391) was purchased from Sigma-Aldrich, St. Louis, MO. Anti-mouse CD152 (CTLA-4) APC (UC10-4B9), anti-mouse Foxp3 PE-Cyanine7 (FJK-16s), anti-mouse CD3e (clone 145-2C11), and anti-mouse CD28 (clone 37.51) were purchased from eBioscience, San Diego, CA. CellTrace™ CFSE Cell Proliferation Kit was purchased from Invitrogen, Carlsbad, CA. ELISA kits for mouse IL-10, TGF-β1, IL-2, IL-4, and IFN-γ were obtained from ExCell Biology Inc., Shanghai, China.

### Cells and cell culture

CD4^+^CD25^+^ Tregs and CD4^+^CD25^−^ T cells were isolated from murine splenocytes by magnetic cell sorting (Miltenyi Biotec, Bergisch Gladbach, Germany), according to the manufacturer’s instructions. CD4^+^CD25^+^ Tregs were isolated using magnetic cell sorting in a two-step procedure. CD4^+^ T cells were pre-enriched by depletion of unwanted cells. CD25^+^ cells were then positively selected from the enriched CD4^+^ T-cell fraction. In brief, non-CD4^+^ T cells were depleted by indirect magnetic labeling with Biotin-Antibody Cocktail (10 μl per 10^7^ cells, 10 minutes at 4 °C) and then incubated with anti-biotin microbeads (20 μl per 10^7^ cells) and CD25-PE (10 μl per 10^7^ cells) for an additional 15 minutes in the dark at 4 °C. The magnetically labeled cell suspension was loaded onto LD Columns placed in the magnetic field of the magnetic cell sorter. The flow-through CD4^+^ cells were collected and centrifuged. The enriched CD4^+^ T-cell fraction were incubated with anti-PE microbeads (10 μl per 10^7^ cells) for 15 minutes in the dark at 4 °C. The flow-through containing CD4^+^CD2^5^ cells were collected after taking cell suspension onto MS Columns. CD4^+^CD25^+^ Tregs were collected after magnetic separation using two MS Columns. The purity of CD4^+^CD25^+^ Tregs was greater than 92% by FACS analysis. Phenotype of these sorted cells was confirmed by Foxp3 staining of a small aliquot, and the sorted cells were found to be enriched with Foxp3^+^ cells. All cell counts were performed on a hemocytometer using trypan blue to exclude dead cells from the counts.

The culture of CD4^+^CD25^+^ Treg was prepared with 10^6^ cells/ml in complete RPMI 1640 medium supplemented with 10% fetal calf serum, 100 U/ml penicillin, 100 μg/ml streptomycin, and 50 μM 2-methoxyestradiol. Anti-CD3 monoclonal antibody (mAb) combined with anti-CD28 mAb at a final concentration of 2 μg/ml was added to the culture for stimulation. In the rhIL-37 stimulation experiments, different concentrations of IL-37 (16 ng/ml, 40 ng/ml, 100 ng/ml or 250 ng/ml) were added to the culture at 12, 24 or 48 hours prior to the suppression assay. In the LPS stimulation experiments, LPS (5 μg/ml) was added to the culture at 3 days before the suppression assay. Methods to stimulate Tregs were summarized in table 1.

After pre-stimulation, cultured CD4^+^CD25^+^ Tregs were used for suppression assay. Another part of cultured cells was used for the marker analysis of CD4^+^CD25^+^ Tregs. Cell culture supernatant was collected for cytokine production assay.

### CFSE staining

CFSE stock solution (5 mM) was prepared in DMSO prior to use. CD4^+^CD25^−^ T cells were washed twice with cold PBS and resuspended (10^6^/ ml) in pre-warmed PBS containing CFSE (5 μM). After incubation (20 minutes, 37 °C, in the dark), staining was stopped by adding five times the staining volume of culture medium (containing 10% fetal bovine serum). Cells were pelleted by centrifugation, resuspended in fresh pre-warmed medium, incubated (10 minutes, 37 °C) to allow the CellTrace™ reagent to undergo acetate hydrolysis, washed twice with PBS, counted and used for *in vitro* suppression assay.

### Suppressive assay

CD4^+^CD25^+^ Tregs were pre-stimulated with rhIL-37 or in combination with LPS before use in the suppressive assay. CD4^+^CD25^+^ Tregs were mixed with CFSE-labeled CD4^+^CD25^−^ T cells in a ratio of 1:4 (the optimal suppressive ratio determined by preliminary experiments) in U-bottom 96-well plates. A total of 3 × 10^4^ cells were suspended in 200 μl of RPMI 1640 medium and stimulated with 2 μg/ml soluble anti-CD3 mAb and 2 μg/ml soluble anti-CD28 mAb in a humidified environment with 5% CO_2_ at 37 °C. After 96 hours of coculture, supernatants were collected for T-cell cytokine production. In parallel, cells were collected and analyzed by flow cytometry to visualize CFSE dilution. Proliferation of CFSE-labeled Tresp was measured by the percentage of CFSE diluting Tresp.

### Flow cytometric analysis

Cultured CD4^+^CD25^+^ Tregs (10^6^) were prepared for the flow cytometric analysis. Tregs were stained by anti-Mouse CD152 (CTLA-4) APC or Armenian Hamster IgG Isotype Control APC as an isotype control. Analytical procedure of Foxp3 was different. After disposing with fresh prepared fixation/permeabilization working solution, samples were washed with permeabilization buffer and incubated with Anti-Mouse Foxp3 PE-Cyanine7 at 4 °C for 30 minutes in the dark. After washing cells, samples were fixed in 1% formaldehyde solution and analyzed by FACSCalibur (BD Biosciences, Mountain View, CA).

### Cytokine measurement by ELISA

At the end of the incubation time, cell supernatants were collected, centrifuged, and stored at −70 °C until tested. IL-10 and TGF-β1 levels in CD4^+^CD25^+^ Treg culture supernatants were measured using ELISA kits, according to the manufacturer’s instructions. To determine T-cell cytokine production, culture supernatants from CD4^+^CD25^+^ Tregs/CD4^+^CD25^−^ T-cell cocultures were collected, and IL-2, IL-4, as well as IFN-γ levels were measured with ELISA. Microplate reader (Spectra MR, Dynex, Richfield, MN) was used to obtain the results.

### Cecal ligation and puncture

For induction of polymicrobial sepsis, mice were subjected to CLP as described previously. In brief, a laparotomy was performed, and the cecum was isolated, ligated below the ileocecal valve, and punctured through with a 23-gauge needle. After removing the needle, a droplet of feces was extruded from both the mesenteric and antimesenteric penetration holes to ensure patency. The peritoneum was closed with fasciae and abdominal musculature by applying simple running sutures. Then, the skin was closed by using simple interrupted sutures. Animals were resuscitated by injecting pre-warmed normal saline (37 °C; 5 ml per 100 g body weight) subcutaneously. Sham operation was performed by isolating the cecum without ligation and puncture. CLP animals were given an intraperitoneally (i.p.) injection of 1 μg rhIL-37 at 2 hours before surgery, 2 hours post surgery, and immediately after surgery, respectively. In the control group, animals were received an identical volume (200 μl) of PBS intraperitoneally at the corresponding time point, which IL-37 was given. Animals were observed for up to 8 days to record the survival rate.

### Statistics analysis

Statistics were performed using SPSS software (SPSS Inc., Chicago, IL). Continuous variables were first tested for normality and equality of variances. The results were expressed as the means ± standard deviation (SD) with the number of independent experiments run in triplicate. One-way ANOVA with post hoc Bonferroni test was used in multiple comparisons. Survival was analyzed with Kaplan-Meier survival curves and compared with the log rank test. Significance was reported at P < 0.05.

## Additional Information

**How to cite this article**: Wang, D.-W. *et al*. Interleukin-37 Enhances the Suppressive Activity of Naturally Occurring CD4^+^CD25^+^ Regulatory T Cells. *Sci. Rep.* **6**, 38955; doi: 10.1038/srep38955 (2016).

**Publisher's note:** Springer Nature remains neutral with regard to jurisdictional claims in published maps and institutional affiliations.

## Figures and Tables

**Table 1:** Methods to stimulate Tregs.

	Methods of stimulation	Time of stimulation
**Treg A**	PBS	24 hours
**Treg B**	IL-37 (16 ng/ml)	24 hours
**Treg C**	IL-37 (40 ng/ml)	24 hours
**Treg D**	IL-37 (100 ng/ml)	24 hours
**Treg E**	IL-37 (250 ng/ml)	24 hours
**Treg F**	PBS	12 hours
**Treg G**	IL-37 (100 ng/ml)	12 hours
**Treg H**	IL-37 (100 ng/ml)	48 hours
**Treg I**	PBS	3 days
**Treg J**	LPS	3 days
**Treg K**	LPS+IL-37 (40 ng/ml)	3 days
**Treg L**	LPS+IL-37 (100 ng/ml)	3 days
**Treg M**	LPS+IL-37 (250 ng/ml)	3 days
